# Patterns of extracellular enzyme activities and microbial metabolism in an Arctic fjord of Svalbard and in the northern Gulf of Mexico: contrasts in carbon processing by pelagic microbial communities

**DOI:** 10.3389/fmicb.2013.00318

**Published:** 2013-10-31

**Authors:** Carol Arnosti, Andrew D. Steen

**Affiliations:** Department of Marine Sciences, University of North Carolina-Chapel Hill, Chapel HillNC, USA

**Keywords:** polysaccharides, glucose metabolism, microbial loop, carbon cycling, enzyme activities, bacterial production, Arctic Ocean, Gulf of Mexico

## Abstract

The microbial community composition of polar and temperate ocean waters differs substantially, but the potential functional consequences of these differences are largely unexplored. We measured bacterial production, glucose metabolism, and the abilities of microbial communities to hydrolyze a range of polysaccharides in an Arctic fjord of Svalbard (Smeerenburg Fjord), and thus to initiate remineralization of high-molecular weight organic matter. We compared these data with similar measurements previously carried out in the northern Gulf of Mexico in order to investigate whether differences in the spectrum of enzyme activities measurable in Arctic and temperate environments are reflected in “downstream” aspects of microbial metabolism (metabolism of monomers and biomass production). Only four of six polysaccharide substrates were hydrolyzed in Smeerenburg Fjord; all were hydrolyzed in the upper water column of the Gulf. These patterns are consistent on an interannual basis. Bacterial protein production was comparable at both locations, but the pathways of glucose utilization differed. Glucose incorporation rate constants were comparatively higher in Svalbard, but glucose respiration rate constants were higher in surface waters of the Gulf. As a result, at the time of sampling ca. 75% of the glucose was incorporated into biomass in Svalbard, but in the northern Gulf of Mexico most of the glucose was respired to CO_2_. A limited range of enzyme activities is therefore not a sign of a dormant community or one unable to further process substrates resulting from extracellular enzymatic hydrolysis. The ultimate fate of carbohydrates in marine waters, however, is strongly dependent upon the specific capabilities of heterotrophic microbial communities in these disparate environments.

## INTRODUCTION

Heterotrophic microbial communities process a considerable proportion of primary productivity in the ocean, and as such are major drivers of the marine carbon cycle (Azam, 1998). In order to access much of this organic matter, members of microbial communities must produce extracellular enzymes of the correct structural specificity to hydrolyze high molecular weight substrates to sizes sufficiently small (ca. 700 Da; Benz, 1990) to be taken into the cell. The ability to produce extracellular enzymes is unevenly distributed among microbes (Zimmerman et al., 2013), but the extent to which enzymatic capabilities vary among entire microbial communities in the ocean is only beginning to be explored. Genomic sequencing has provided information about the potential capabilities of an increasing number of organisms (e.g., Glöckner et al., 2003; Bauer et al., 2006; Weiner et al., 2008; Martinez-Garcia et al., 2012), but these organisms represent only a small fraction of marine microbes. Metagenomic sequencing has yielded considerable insight into community enzymatic potential (Cottrell et al., 2005; Gomez-Pereira et al., 2012), but provides no information about the extent to which this potential is expressed in the ocean.

To measure directly the abilities of heterotrophic microbial communities to produce specific extracellular enzymes, we compared hydrolysis of structurally distinct polysaccharides at different depths and locations in the ocean (e.g., Arnosti et al., 2005, 2009; Arnosti, 2008; Steen et al., 2008). These polysaccharides (pullulan, laminarin, xylan, fucoidan, arabinogalactan, and chondroitin sulfate) are derived from marine organisms. Moreover, enzymes hydrolyzing these polysaccharides have been identified in marine bacteria, and/or genes corresponding to the enzymes hydrolyzing these polysaccharides have been identified in the genomes of fully sequenced marine bacteria (e.g., Glöckner et al., 2003; Bauer et al., 2006; Alderkamp et al., 2007; Weiner et al., 2008). Comparison of a decade’s worth of data obtained across a broad range of surface-ocean sites revealed that pelagic microbial communities at high latitudes can enzymatically hydrolyze, and thus gain access to, a narrower spectrum of polysaccharide substrates than their temperate counterparts (Arnosti et al., 2011). This pattern parallels latitudinal gradients in microbial diversity, with reduced community diversity toward the poles (Pommier et al., 2007; Fuhrman et al., 2008). We thus have evidence of latitudinal differences in microbial function that coincide with data on community diversity. The Arctic fjords of Svalbard, where a limited range of polysaccharide substrates is typically hydrolyzed, and the northern Gulf of Mexico, where all of the polysaccharide substrates measured to date have been hydrolyzed, provide contrasting end members for this pattern (Arnosti et al., 2011).

Extracellular enzymatic hydrolysis, however, is only the initial step in heterotrophic carbon cycling: subsequent steps in metabolic pathways determine the ultimate fate of substrate carbon: remineralization, incorporation into biomass, or transformation and excretion as DOC (dissolved organic carbon). We hypothesized that differences in patterns of polysaccharide hydrolysis may be reflected in downstream differences in carbohydrate uptake and metabolism. To test this hypothesis, in the present study we investigated surface and subsurface patterns of enzymatic hydrolysis, glucose uptake, and respiration, and bacterial biomass production in an Arctic fjord of Svalbard (Smeerenburg Fjord). We compare these results to those obtained in our previous investigation in the northern Gulf of Mexico (Steen et al., 2012). This comparison of Arctic and Gulf of Mexico end members is particularly relevant because they are the only sites for which we have multi-year data on enzyme activities in the water column (e.g., Arnosti, 2008; Arnosti et al., 2009, 2011), enabling us to compare our data on microbial metabolism within a framework of robust differences in enzymatic hydrolysis patterns. By comparing data on enzyme activities with steps further along heterotrophic metabolic pathways, we can begin to determine the fate of organic carbon initially made available to microbial communities through the activities of extracellular enzymes.

## MATERIALS AND METHODS

### STUDY SITES AND SAMPLE COLLECTION

#### The northern Gulf of Mexico

Water was collected at four depths (2, 17, 150, and 280 m) at Station 2 (28° 32.041′N, 89° 21.503′W; water column depth 285 m), and at six depths (2, 45, 125, 350, 700, and 905 m) at Station 3 (28° 16.369′N, 89° 21.772′W, water column depth 925 m) from the R/V *Cape Hatteras* on September 27th and 28th 2007, using Niskin bottles mounted on a CTD-equipped rosette (Steen et al., 2012). Samples were processed for all measurements immediately aboard ship. Note that data from the Gulf of Mexico, with the exception of the cell counts, have been previously reported in Steen et al. (2012).

#### Svalbard

Seawater (surface water: 2 m, T = 3.3°C; bottom water: 205 m, T = 1.5°C) was collected via Niskin bottle at Station J (79° 42.8′N, 011° 05.2′E, 220 m water column depth) in Smeerenburg Fjord, Svalbard, on 15 August 2008. Water was stored in triple-rinsed plastic carboys, and stored in coolers filled with surface water at approximately in situ temperature for approximately 12 h during transit to the laboratory at Ny Ålesund. The transport time likely did not substantially influence enzyme activities or bacterial production, since extracellular enzymes in Svalbard surface waters are stable over timescales of 24–36 h (Steen and Arnosti, 2011). The observation that radiotracer measurements were linear over 24 h of incubation in the dark suggests that organic matter production and consumption were not so tightly coupled that a 12-transit under approximately in situ light and temperature conditions would have a major effect on measured rates.

### EXTRACELLULAR ENZYMATIC HYDROLYSIS RATES

Hydrolysis rates of six different polysaccharides that had been labeled with fluoresceineamine (FLA; Sigma, Isomer II) were measured by the method of Arnosti (1995, 2003). These polysaccharides (pullulan, laminarin, xylan, fucoidan, arabinogalactan, and chondroitin sulfate; all from Sigma) differ in monomer composition and linkage position. Pullulan is α(1,6) linked-maltotriose [α(1,4) glucose], laminarin is β(1,3) glucose, xylan is a β(1,4) polymer of xylose, fucoidan is a sulfated fucose-containing polysaccharide, arabinogalactan is a mixed polymer of arabinose and galactose, and chondroitin sulfate is a sulfated polymer of galactoseamine and glucuronic acid (β-GlcA(1,3)-GalNAc(1,4)). These polysaccharides were selected as substrates because carbohydrates constitute a considerable fraction of marine organic matter (Hedges et al., 1988; Benner et al., 1992), activities of enzymes hydrolyzing these polysaccharides have been widely measured in marine waters and sediments (Arnosti et al., 2005; Arnosti, 2008; Teske et al., 2011), and most are components of marine algae and phytoplankton (Painter, 1983; Alderkamp et al., 2007).

To measure enzymatic hydrolysis rates in seawater, FLA-polysaccharides were added to 50 mL water samples to a final concentration of 3.5 μmol monomer L^-^^1^ (2.8 μmol monomer L^-^^1^ in the case of xylan). These 50 ml samples were divided into three replicate incubations of ~17 ml each. FLA-polysaccharides were also added at the same concentrations to a killed control consisting of a single replicate of autoclaved seawater. Samples were incubated at 4°C [the temperature of the cold room available in the lab on Svalbard; sample incubations were made at temperatures corresponding to *in situ* for the Gulf of Mexico samples, see Steen et al. (2012) for details]. Each incubation was sub-sampled immediately after the addition of polysaccharides and again at 3, 7, 10, and 15 days of incubation (the 15 days samples for xylan from Svalbard were lost in transport). Maximum rates reported here are from 15 days (10 days for xylan). To subsample the incubations, ca. 2 ml of the incubation was withdrawn via sterile syringe and filtered through 0.2 μm pore-size surfactant-free cellulose-acetate syringe filters into combusted glass vials, which were immediately capped and frozen until analysis. Frozen samples were thawed, diluted, and injected on a gel permeation chromatography system with a fluorescence detector set to excitation and emission maxima of 490 and 530 nm, respectively. Hydrolysis rates were calculated from the systematic changes in substrate molecular weight with incubation time, as described in detail in Arnosti (2003). Note that the data for surface-water enzyme activities from Station J was included in Arnosti et al. (2011).

### BACTERIAL PRODUCTION

Bacterial productivity was determined from the rate of incorporation of ^3^H-labeled leucine measured in Svalbard by the method of Kirchman (1993). L-[3,4,5 ^3^H(N)]-leucine (Perkin–Elmer) was diluted 1:20 with non-radioactive L-leucine to a specific activity of 5.77 Ci mmol^-^^1^. 150 pmol total leucine was added to glass scintillation vials to which 15 ml seawater was added. Killed controls (one per sample source) were amended with 835 μL 100% trichloroacetic acid (TCA) prior to the addition of seawater. Triplicate samples for each water source were incubated for 3 h. The incubation was stopped by the addition of 835 μL 100% TCA. Vials were then heated to 80°C in a heating block for 15 min to precipitate proteins, and air-cooled. The precipitate was then filtered onto 0.2 μm, 25 mm diameter nitrocellulose filters shortly thereafter. The sample vials and filter tower were rinsed twice with 3 ml ice-cold 5% TCA, twice with 3 ml, ice-cold ethanol, and then the tower was removed and the filter was rinsed with 1 ml cold ethanol. The nitrocellulose filters were then transferred to new glass scintillation vials, dissolved in 0.5 ml ethyl acetate, and mixed with 10 ml ScintiSafe. Vials were allowed to “rest” at room temperature for 8 days to maximize counting efficiency, and then radio-assayed.

### ASSIMILATION AND RESPIRATION OF GLUCOSE

Glucose assimilation rates were determined from the rate of incorporation of ^14^C-labeled glucose into particulate organic carbon (POC). Remineralization was determined from the rate of production of radiolabeled dissolved inorganic carbon (DIC), using a modification of the method of Hobbie and Crawford (1969). Uniformly ^14^C-labeled glucose (7.5 pmol; Perkin–Elmer, 200.6 mCi mmol^-^^1^) was added to 15 ml seawater in 20 ml glass scintillation vials with plastic caps for a final concentration of 1 nmol L^-^^1^ glucose. TCA (100%) was added to killed controls, prior to addition of seawater, to a final concentration of 5%. Since the level of activity to be expected in these waters was unknown a priori, samples for both PO^14^C and DI^14^C measurement were incubated at 4°C and triplicate live incubations plus one killed control were sampled after 3, 8, and 24 h incubation. Because glucose respiration rate constants and glucose assimilation constants showed no consistent trends with incubation time, rates reported here are the average and standard deviation of all samples at all time points (i.e., nine live incubations and three killed controls).

Glucose incorporation and remineralization rate constants and glucose utilization efficiencies were measured on Svalbard as described in detail in Steen et al. (2012), with the exception that the CO_2_ trapping/scintillation cocktail consisted of 40% ScintiSafe 3 (Fisher), 25% β-phenethylamine, and 35% methanol. Trapping efficiency of this solution measured with NaH^14^CO_3_ added to seawater was 105 ± 4% compared to radiolabeled bicarbonate added directly to the trapping cocktail.

Glucose assimilation and remineralization rate constants were calculated assuming first-order kinetics, as

$$k=\frac{1}{t}\ln\left(\frac{a_{glu,}to}{a_{glu,}to-a_{t}}\right)$$

where *a*_t_ is the activity of PO^14^C or DI^14^C, *a*_glu,t0_ is the initial activity of ^14^C-glucose added to the incubation, and *t* is incubation time.

Glucose utilization efficiency was calculated as the rate constant of glucose assimilation divided by the sum of glucose assimilation and respiration rate constants; it reflects the extent to which the glucose that is taken up by a cell is incorporated into biomass.

*Cell counts*. Cell counts were carried out on samples from the Gulf of Mexico; no samples were collected for cell counts from Svalbard in 2008. Ten milliliter of seawater samples from each depth were fixed with 0.2 μm filtered formaldehyde (2% v/v final concentration) and stored at 4°C until further treatment. One slide per depth was prepared by staining 2–5 ml of samples with 4′, 6-diamidino-2-phenylindole (DAPI, 0.1 mgl^-^^1^ final concentration), using the method of Porter and Feig (1980). Cells were microscopically examined using an epifluorescence microscope (Olympus, magnification ×1000) equipped with a digital camera (Olympus TH4-100); 20 pictures or at least 1000 cells were counted per sample.

## RESULTS

### EXTRACELLULAR ENZYMATIC HYDROLYSIS

**FIGURE 1:**
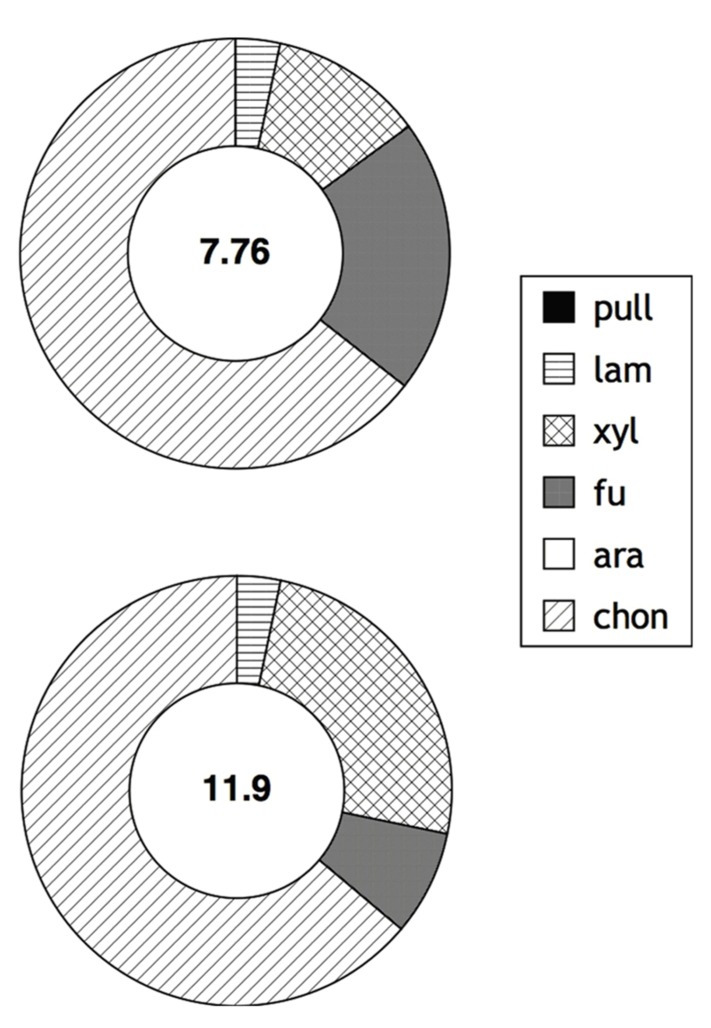
**Enzymatic hydrolysis rates of polysaccharides in surface and bottom waters (2 and 205 m, respectively; top and bottom circles) of Station J in Smeerenburg Fjord, Svalbard in 2008.** Number in center of circle corresponds to summed maximum hydrolysis rate (nmol monomer L^-^^1^ h^-^^1^) of enzyme activities. Shading of circle shows relative contribution of each activity to the total measured. Pull, pullulan; lam, laminarin; xyl, xylan; fu, fucoidan; ara, arabinogalactan; chon, chondroitin sulfate. Note that surface water data was initially reported in Arnosti et al. (2011).

Hydrolysis of fluorescently labeled polysaccharides over timescales of days to weeks provides information about the potential of a community to respond enzymatically to the addition of specific substrates, rather than reflecting the activity and state of a microbial community at the time of sample collection. The measurements made with FLA-polysaccharides therefore integrate microbial responses to substrate addition; such responses potentially include microbial growth and shifts in population structures, as well as induction of specific enzymes. In surface as well as bottom water of Smeerenburg Fjord, four of the six polysaccharide substrates – laminarin, xylan, fucoidan, and chondroitin sulfate – were hydrolyzed after 15 days; pullulan and arabinogalactan were not (**Figure 1**). Chondroitin was hydrolyzed most rapidly in surface water, followed by fucoidan, xylan, and then laminarin. In bottom water, chondroitin was also hydrolyzed most rapidly, followed by xylan, fucoidan, and laminarin. Maximum hydrolysis rates of chondroitin and xylan were more rapid in bottom water than in surface water; maximum hydrolysis rates of laminarin and fucoidan were comparable at both depths. Summed hydrolysis rates (the sum of the maximum rate observed for hydrolysis of each substrate) were 7.8 nmol monomer L^-^^1^ h^-^^1^ for surface water, and 11.9 nmol monomer L^-^^1 ^h^-^^1^for bottom water.

**FIGURE 2:**
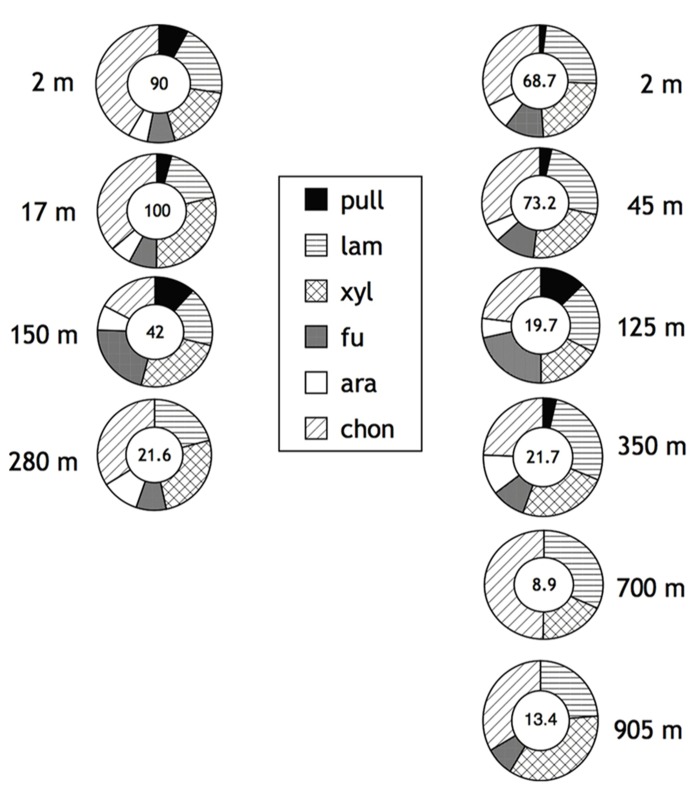
**Enzymatic hydrolysis rates of polysaccharides in depth profiles of Stations 2 (left) and 3 (right) in the northern Gulf of Mexico (see Materials and Methods for specific location).** Water column depth shown to the left of Stn. 2 samples, to the right of Stn. 3 samples. Number in center of circle corresponds to summed maximum hydrolysis rate (nmol monomer L^-^^1^ h^-^^1^) of enzyme activities. Shading of circle shows relative contribution of each activity to the total measured. Pull, pullulan; lam, laminarin; xyl, xylan; fu, fucoidan; ara, arabinogalactan; chon, chondroitin sulfate. Data replotted from Steen et al. (2012).

The pattern of substrates hydrolyzed observed in Smeerenburg Fjord contrasts strongly with that measured in the northern Gulf of Mexico. In the Gulf of Mexico, all six substrates were hydrolyzed in the upper water column (surface through a depth of 150 m for Stn. 2, surface through a depth of 250 m for Stn. 3), only at the deepest stations (280 m at Stn. 2; 700 and 905 m for Stn. 3) were only five (Stn. 2), or four (Stn. 3, 905 m), or three (Stn. 3, 700 m) substrates hydrolyzed. In general, maximum rates of laminarin, xylan, and chondroitin hydrolysis were considerably more rapid than rates of pullulan, fucoidan, and arabinogalactan hydrolysis (**Figure 2**). Summed hydrolysis rates ranged from 21 to 100 nmol monomer L^-^^1^ h^-^^1^ at Stn. 2 and 8.9 to 73.2 nmol monomer L^-^^1^ h^-^^1^ at Stn. 3. Summed hydrolysis rates were lower deeper in the water column.

### LEUCINE INCORPORATION

^3^H-leucine uptake in Smeerenburg Fjord was 36.9 ± 11.3 pmol L^-^^1^ h^-^^1^ in surface water, and 11.8 ± 1.5 pmol L^-^^1^ h^-^^1^ in bottom water (**Table 1**). In the Gulf of Mexico at Stn. 2, leucine uptake (measured at single timepoints) was 76 pmol L^-^^1^ h^-^^1^ in surface water, dropping to 39 pmol leucine L^-^^1^ h^-^^1^ at 17 m, 0.7 pmol L^-^^1^ h^-^^1^ at 150 m, and below the detection limit at 285 m. At Stn. 3, leucine uptake was 22, 7.3, and 0.8 pmol L^-^^1^ h^-^^1^ at the surface, 47 and 125 m, respectively, and below the detection limit at 700 and 905 m (**Table 1**).

**Table 1:** Water column sample depths, temperature, leucine incorporation, glucose incorporation and respiration rate constants, and cell counts (Gulf of Mexico only) for Station J, Smeerenburg Fjord, Svalbard, in August 2008, and for Stns. 2 and 3 in the northern Gulf of Mexico in September 2007.

Station; sample depth	*In situ* water T(°C)	Leucine incorporation (pmol L^-^^1^ h^-^^1^)	Glucose respiration rate constant (×10^-^^3^ h^-^^1^)	Glucose incorporation rate constant (×10^-^^3^ h^-^^1^)	Glucose utilization efficiency (% into biomass)	Cell counts (×10^6^ ml^-^^1^)
**Smeerenburg Fjord**
2 m	3.3	36.9 ± 11.3	7.0 ± 0.1	19 ± 0.28	73	n.s.
205 m	1.5	11.8 ± 1.5	4.4 ± 0.84	14 ± 0.57	76	n.s.
**Gulf of Mexico Station 2**
2 m	29	76 ± 63	40.6 ± 2.3	48	54	6.3
17 m	29	39 ± 50	32.3 ± 1.7	29	48	1.9
150 m	16	0.7 ± 0.2	1.8 ± 0.43	2.1	54	n.a.
285 m	11	n.d.	1.7 ± 0.50	1.2	42	n.a.
**Gulf of Mexico Station 3**
2 m	29	22 ± 10	20.5 ± 1.1	14	41	n.a.
47 m	25	7.3 ± 2.4	6.1 ± 0.11	4.2	41	6.3
125 m	18	0.8 ± 0.2	2.1 ± 0.33	1.3	39	2.9
350 m	9.8	n.d.	1.5 ± 0.18	0.6	28	1.2
700 m	6.4	n.d.	1.7 ± 0.21	0.6	26	1.3
905 m	5.4	n.d.	1.3 ± 0.16	0.7	33	1.8

*
Data from the Gulf of Mexico, with the exception of the cell counts, are from Steen et al. (2012). n.a., not available (samples lost); n.d., not detected; n.s., no samples collected for this analysis.*

### GLUCOSE METABOLISM

In Smeerenburg Fjord as well as in the northern Gulf of Mexico, rate constants for glucose respiration and for glucose assimilation were higher in surface than in subsurface waters, but the relative difference between rate constants measured in surface and in subsurface waters differed between locations (**Table 1**). In Smeerenburg Fjord, rate constants for glucose respiration and glucose incorporation decreased by approximately 37 and 26%, respectively, between surface and bottom water, while over a comparable depth range in the northern Gulf of Mexico, these rate constants decreased by 96 and 90%, respectively (**Table 1**). The relative magnitude of rate constants for assimilation and respiration also differed between locations. In Smeerenburg Fjord, the rate constant for glucose assimilation was two to three times greater than the rate constant for glucose respiration. In the northern Gulf of Mexico, in contrast, with two exceptions, rate constants for glucose respiration were greater than for assimilation (**Table 1**). The glucose respiration rate constants in surface and near-surface waters in the northern Gulf of Mexico were higher than in Smeerenburg Fjord surface water; in subsurface waters (below 125–150 m), rate constants for respiration were lower than for bottom water in Smeerenburg Fjord. Rate constants for glucose incorporation, in contrast, were generally higher in Smeerenburg Fjord than in the northern Gulf of Mexico, with the exception of samples from the surface and from 17 m at Stn. 2 (**Table 1**).

These measurements of rate constants for glucose respiration as well for glucose incorporation permit calculation of glucose utilization efficiency, i.e., the fraction of glucose incorporated that is converted into biomass. This quantity is conceptually similar to bacteria growth efficiency, in that it relates the quantity of glucose catabolized to the total quantity taken up by microorganisms. However, whereas bacterial growth efficiency reports the metabolic fate of all carbon taken up by microorganisms, glucose utilization efficiency reflects only the metabolic fate of glucose, which may differ from that of bulk organic carbon (see Discussion). These values in Smeerenburg Fjord (73 and 76% for surface and bottom waters, respectively) were considerably greater than for the northern Gulf of Mexico (42–54% at Stn. 2, 26–41% at Stn. 3; **Table 1**).

### CELL COUNTS

Cell counts were made only for the northern Gulf of Mexico. These counts ranged from 1.2–1.9 × 10^6^ cell ml^-^^1^ to 6.3 × 10^6^ cell ml^-^^1^; samples lost in transport are marked as “not available” (**Table 1**).

## DISCUSSION

### ENZYMATIC HYDROLYSIS RATES AND PATTERNS

**FIGURE 3:**
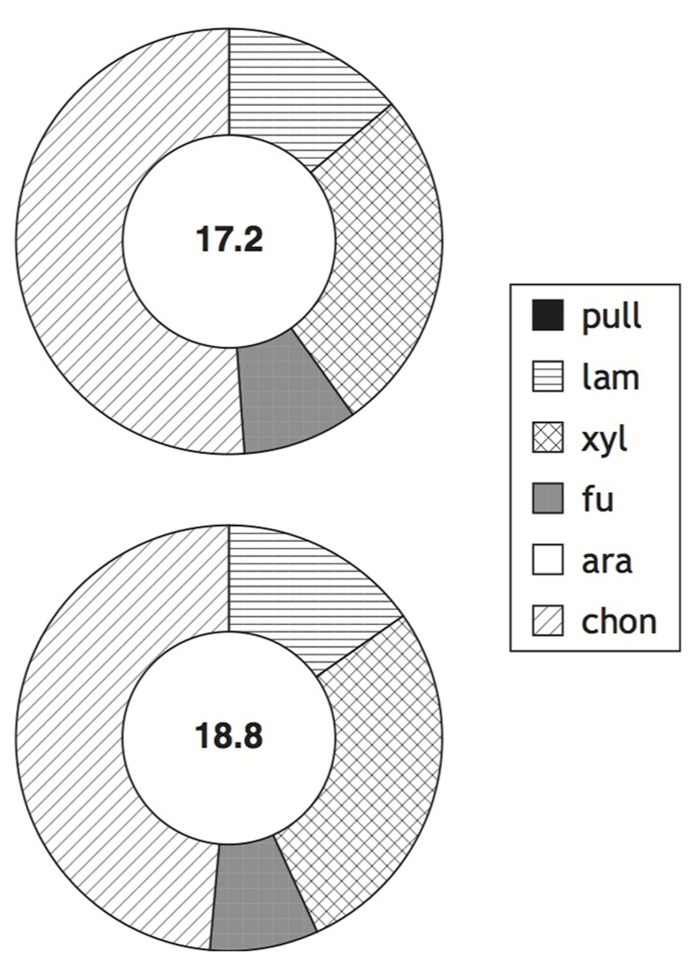
**Enzymatic hydrolysis rates of polysaccharides in surface and bottom waters (2 and 211 m, respectively; top and bottom circles) of Station J in Smeerenburg Fjord, Svalbard in 2007.** Number in center of circle corresponds to summed maximum hydrolysis rate (nmol monomer L^-^^1^ h^-^^1^) of enzyme activities. Shading of circle shows relative contribution of each activity to the total measured. Pull, pullulan; lam, laminarin; xyl, xylan; fu, fucoidan; ara, arabinogalactan; chon, chondroitin sulfate. Data replotted from Teske et al. (2011).

The ability to produce specific extracellular enzymes varies substantially among individual organisms, as demonstrated by microbiological, molecular biological, and genomic investigations (e.g., Martinez et al., 1996; Glöckner et al., 2003; Bauer et al., 2006; Alderkamp et al., 2007; Wegner et al., 2013). The extent to which the enzymatic capabilities of entire microbial communities may vary spatially and temporally, however, is only beginning to be explored (Allison et al., 2012; Arnosti et al., 2012; Gomez-Pereira et al., 2012; Teeling et al., 2012). Our recent work has demonstrated that there is a latitudinal gradient in enzymatic activities in surface marine waters, with a reduced spectrum of activities at higher latitudes (Arnosti et al., 2011). The northern Gulf of Mexico and Smeerenburg Fjord represent opposite ends of this spectrum, with hydrolysis of all substrates in surface waters and the upper water column at both stations in the northern Gulf of Mexico, and only four of six substrates hydrolyzed in surface and bottom waters of Smeerenburg Fjord (**Figures 1** and **2**). A similar pattern was observed at Smeerenburg Fjord in 2007 (**Figure 3**; Teske et al., 2011). These measurements, plus our measurements in 1999 and 2001 in surface waters of Smeerenburg Fjord (Arnosti et al., 2005; Arnosti, 2008) suggest that patterns of enzyme activities are repeatable over multiple summers in Smeerenburg Fjord. In the northern Gulf of Mexico, patterns of enzyme activities in surface waters likewise are reproducible over several years. As noted in Arnosti et al. (2011), two surface water stations close to Stns. 2 and 3, sampled in 2001, also demonstrated hydrolysis of all six substrates, at maximum rates very similar to those measured at Stns. 2 and 3. Additionally, inshore waters of the Gulf of Mexico, sampled in 2006, also showed hydrolysis of all six polysaccharides in surface waters at rates comparable to those measured at Stns. 2 and 3 (Arnosti et al., 2009).

The comparative stability of these patterns at each location over the years may reflect stability in seasonal microbial community composition, which have been shown in other locations to exhibit repeating annual patterns (e.g., Fuhrman, 2009; Gilbert et al., 2012; Grzymski et al., 2012). The contrasting hydrolysis patterns between Smeerenburg Fjord and the northern Gulf of Mexico may be due to the major compositional differences between pelagic microbial communities of Arctic and temperate environments (Bano and Hollibaugh, 2002; Pommier et al., 2007; Fuhrman et al., 2008; Ghiglione et al., 2012), and to differences in function correlated with compositional differences (Arnosti et al., 2012).

### BACTERIAL PROTEIN PRODUCTION

In contrast to measurements of enzymatic hydrolysis using fluorescently labeled polysaccharides, which represent the potential of a community to respond to a substrate over comparatively long timescales, measurements of microbial leucine incorporation are made on timescales of a few hours, more closely reflecting the activities and capabilities of microbial communities at the time of sampling. From this perspective, in Smeerenburg Fjord the measurements of bacterial production reveal differences between surface and bottom water that are not evident from the enzymatic hydrolysis rates and patterns. Leucine incorporation was approximately three times greater in surface than in bottom waters (**Table 1**), suggesting greater microbial community protein production in surface relative to bottom waters. Some of the difference between surface and bottom waters could be due to differences in microbial population size. Although no information on cell counts is available from 2008, in 2010 measurements of surface and bottom water in this same fjord and station showed a factor of 3 difference in cell counts (5.6 and 1.7 × 10^-^^5^ cells ml^-^^1^ in surface and bottom waters, respectively), and a factor of 10 difference in leucine incorporation (Steen and Arnosti, 2013). Bacterial production measured in 2010 was ca. 79 pmol leucine L^-^^1^ h^-^^1^ in surface water, approximately twice the rate measured in 2008, and ca. 7 pmol leucine L^-^^1^ h^-^^1^ in bottom water (Steen and Arnosti, 2013), approximately two-thirds of the rate measured in 2008. Differences in bulk (and likely also in cell-specific) leucine incorporation rates in Svalbard in both 2008 and 2010 thus point to bottom water microbial communities that are less actively growing than their surface-water counterparts.

The range of leucine incorporation rates measured in Smeerenburg Fjord are in any case comparable to values measured for surface water in other locations, including an average of ca. 28 pmol leucine L^-^^1^ h^-^^1^ for a range of Arctic locations in the summer (Nikrad et al., 2012), and values of 45 pmol leucine L^-^^1^ h^-^^1^ for the open Kara Sea (Meon and Amon, 2004). Profiles from a number of stations in the Kara Sea also showed depth-related decreases in leucine incorporation (Rich et al., 1997) similar in proportion to those we observed.

In the northern Gulf of Mexico, leucine incorporation rates – as well as cell counts – also decreased with water column depth. The decrease in leucine incorporation was greater than the decline in cell counts, again pointing to subsurface microbial communities that were growing less actively than the surface communities (**Table 1**). A similar pattern of a greater declines in leucine incorporation compared to cell counts was also observed at a site further south and west in the Gulf of Mexico (Skoog et al., 1999). In general, leucine incorporation rates in the northern Gulf of Mexico were comparable to those measured in Smeerenburg Fjord.

The observation that bacterial leucine incorporation rates were similar at the stations in the northern Gulf of Mexico and in Smeerenburg Fjord, despite the many physical and biological differences between these environments, is in accordance with a recent cross-system review of bacterial activity in polar and temperate environments, which demonstrated that there is little systematic difference in bacterial production in a range of environments across a temperature range of ca. 4–30°C (Kirchman et al., 2009). Although the *in situ* temperature at the time of sampling in Smeerenburg Fjord was slightly lower than 4°C, the waters of this fjord during the late summer typically are within a range of 2.5–5°C (Arnosti, 2008; Teske et al., 2011; Steen and Arnosti, 2013), and our samples were all incubated at 4°C. The observation that bacterial production rates are not a simple function of environmental temperature is also demonstrated by the fact that bacterial protein production in bottom water in Smeerenburg Fjord greatly exceeded most of the sub-surface rates measured in the northern Gulf of Mexico, despite the fact that the *in situ* temperatures at most of the depths sampled in the Gulf were considerably above the *in situ* and incubation temperatures of the Svalbard samples.

### GLUCOSE METABOLISM

Glucose respiration and incorporation were measured in order to assess ability of microbial communities to incorporate carbohydrates that could be made available for uptake via the activity of extracellular enzymes. Although the ability to take up and metabolize different monosaccharides varies among individual organisms, focusing on glucose metabolism is a practical first step in determining the fate of low molecular weight carbohydrates in ocean waters: glucose is the most commonly detected monosaccharide in marine systems (e.g., Rich et al., 1996; Skoog et al., 2002), including the Gulf of Mexico (Skoog et al., 1999), and is a major constituent of combined carbohydrates as well (Cowie and Hedges, 1984). This focus is also relevant for Svalbard, as pelagic microbial communities in Svalbard have been shown to respond strongly to glucose addition, doubling in cell numbers in short periods of time (Topper et al., 2010).

The rate constants for glucose incorporation in Smeerenburg Fjord (**Table 1**) are equivalent to or at the upper end of those reported from other high latitude locations. At a station at 79°N, Rich et al. (1997) reported glucose incorporation rate constants of ca. 0.38 d^-^^1^ in surface waters and ca 0.1 day^-^^1^ at 40 m depth (our values are equivalent to 0.63 and 0.42 day^-^^1^, for surface and bottom waters, respectively). In the western Arctic (Beaufort and Chuckchi Seas), summer average value were found to be approximately 0.025 h^-^^1^ (Nikrad et al., 2012).

The range of rate constants for glucose incorporation in the northern Gulf of Mexico (equivalent to 1.1–0.03 day^-^^1^ at Stn. 2, and 0.34–0.016 day^-^^1^ at Stn. 3) were also comparable to or somewhat higher than other reported values. In a Pacific transect from ca. 12°South to 12°North along 140°West, rate constants for glucose incorporation varied by station and season from ca. 0.1 day^-^^1^ to ca. 0.6 day^-^^1^, with average values of 0.26 ± 0.2 day^-^^1^ north of the equator and 0.43 ± 0.02 day^-^^1^ south of the equator (Rich et al., 1996). At a site south and west of Stns. 2 and 3 in the Gulf of Mexico, rate constants for glucose incorporation (estimated from plots of glucose concentrations and uptake rates) spanned a range of ca. 0.03–0.67 day^-^^1^ (Skoog et al., 1999).

A comparison of glucose metabolism in Smeerenburg Fjord and in the northern Gulf of Mexico brings to light differences in microbial carbon processing in these locations, measured over short-term incubations, that were not evident from measurements of microbial protein production. In particular, the comparatively high values (73–76%) for glucose incorporation in Smeerenburg Fjord are at the upper end of values reported in a number of other studies, including glucose utilization efficiencies of 40–70% across an equatorial transect at 140°West (Rich et al., 1996), and average glucose utilization efficiency of 67% for a range of stations in the Mediterranean, northeast Atlantic, and English Channel (Williams, 1970). Multiple measurements of glucose utilization efficiency at a range of seasons and sites in the western Arctic and sub-Arctic, however, also showed comparatively high median utilization efficiencies of 62–73% (Griffiths et al., 1984).

### CARBOHYDRATE ACCESS AND UTILIZATION IN SMEERENBURG FJORD AND THE NORTHERN GULF OF MEXICO

A comparison of glucose utilization efficiency in Smeerenburg Fjord and the northern Gulf of Mexico suggests that the fate of glucose-derived carbon at the time of sampling differed strikingly in these two environments: much of the glucose was incorporated into biomass in Smeerenburg Fjord, whereas the majority was respired to CO_2_ in the northern Gulf of Mexico. This difference was not due to lower overall metabolic activity of the microbial community in Smeerenburg Fjord, as demonstrated by leucine incorporation (**Table 1**). Moreover, the observation that glucose incorporation and respiration rate constants did not change systematically with increasing incubation time (see Materials and Methods) suggests that glucose incorporation rate constants measured in Smeerenburg Fjord are not simply a reflection of insufficient time to respire carbon that has been taken into the cell. The limited spectrum of enzyme activities measurable in Svalbard waters (**Figure 1**) therefore does not indicate an inactive (as measured by leucine incorporation) or inefficiently functioning (as measured by glucose incorporation) pelagic microbial community. Instead, these results may point to a high degree of enzymatic specialization and efficient use of available resources among members of the pelagic communities of Svalbard.

This efficiency and specialization likely reflects the distinct capabilities of different subfractions of the microbial community that use glucose or leucine, or produce extracellular enzymes in pelagic waters. Efficient incorporation of glucose into biomass, for example, should not be equated with overall efficiency in incorporation of bulk dissolved organic matter intobiomass. Little correlation was found between bacterial biomass production as measured by leucine uptake and by glucose uptake in Arctic waters (Rich et al., 1997). This disconnect is probably due to the fact that glucose is likely used as a substrate by a much smaller fraction of the microbial community than is leucine, as was recently reported in investigations in the western Arctic and coastal Canadian Arctic (Kirchman et al., 2007; Alonso-Saez et al., 2008). Glucose uptake in the Arctic may vary by season, location, and specific group of organisms (e.g., Alonso-Saez et al., 2008, 2012; Nikrad et al., 2012). The processing and fate of glucose in Svalbard and in the northern Gulf of Mexico thus likely reflect the distinct metabolic capabilities of specific subfractions of heterotrophic microbial communities.

In a similar manner, the activities of extracellular enzymes measured in Svalbard and in the Gulf of Mexico likely also reflect fractions of the microbial community that differ in their response to substrate input. In the western Arctic, a higher percentage of cells took up EPS (exopolymeric substances, which are typically carbohydrate-rich) than glucose, with the absolute percentages varying depending on station location (Elifantz et al., 2007). In the Delaware estuary, in contrast, a higher percentage of cells incorporated glucose than EPS (Elifantz et al., 2005). Moreover, some of the organisms taking up monosaccharides are not necessarily those that have produced the extracellular enzymes. Members of the SAR11, for example, are not likely candidates for production of extracellular enzymes (Giovannoni et al., 2005), although they may take up substantial quantities of glucose (e.g., Nikrad et al., 2012), whereas members of the *Bacteroidetes* have been shown to consume high molecular weight substrates (Kirchman, 2002) and contain genes for polysaccharide hydrolysis (Bauer et al., 2006; Gomez-Pereira et al., 2012), yet may be comparatively underrepresented in glucose uptake (e.g., Elifantz et al., 2005; Alonso-Saez et al., 2008, 2012).

Multiple groups of heterotrophic microbes – some with overlapping membership, some distinct from one another – thus likely contribute to polysaccharide hydrolysis and to consumption of resultant hydrolysis products in pelagic marine waters. The observation that the spectrum of substrates hydrolyzed by microbial enzymes is reproducible over several years in specific locations points to stability in functional specialization in these environments. The ability to produce specific extracellular enzymes, given sufficient response time, appears to be a capability of microbial communities that does not scale directly with shorter-timescale measurements of bacterial protein production.

The extent to which the fate of polysaccharide hydrolysis products varies by monomer composition, nutrient conditions, or season (Rich et al., 1996) requires further investigation. Nonetheless, our data demonstrates that a location characterized by a narrower spectrum of enzyme activities does not show indications of a notably inactive or inefficiently functioning pelagic microbial community compared to locations and depths characterized by a broader spectrum of enzyme activities. The observation that glucose metabolism in the northern Gulf of Mexico at the time of sampling was dominated by respiration to CO_2_, while most of the glucose taken up by pelagic microbes in Svalbard was incorporated into biomass, however, suggests that the pathways by which the same carbohydrates are cycled can be quite different. The ultimate fate of marine carbohydrates in the ocean depends on the enzymatic and metabolic capabilities of specific subfractions of heterotrophic microbial communities. In light of predictions for changes in Arctic microbial communities and carbon processing as an outcome of environmental change (Kirchman et al., 2009), the balance of carbon incorporation and respiration (as well as the nature of carbohydrates enzymatically available to microbial communities) may change substantially in the future.

## Conflict of Interest Statement

The authors declare that the research was conducted in the absence of any commercial or financial relationships that could be construed as a potential conflict of interest.
